# Colocalization of Protein Kinase A with Adenylyl Cyclase Enhances Protein Kinase A Activity during Induction of Long-Lasting Long-Term-Potentiation

**DOI:** 10.1371/journal.pcbi.1002084

**Published:** 2011-06-30

**Authors:** Myungsook Kim, Alan Jung Park, Robbert Havekes, Andrew Chay, Leonardo Antonio Guercio, Rodrigo Freire Oliveira, Ted Abel, Kim T. Blackwell

**Affiliations:** 1 George Mason University, The Krasnow Institute for Advanced Studies, Fairfax, Virginia, United States of America; 2 University of Pennsylvania, Department of Biology, Philadelphia, Pennsylvania, United States of America; Université Paris Descartes, Centre National de la Recherche Scientifique, France

## Abstract

The ability of neurons to differentially respond to specific temporal and spatial input patterns underlies information storage in neural circuits. One means of achieving spatial specificity is to restrict signaling molecules to particular subcellular compartments using anchoring molecules such as A-Kinase Anchoring Proteins (AKAPs). Disruption of protein kinase A (PKA) anchoring to AKAPs impairs a PKA-dependent form of long term potentiation (LTP) in the hippocampus. To investigate the role of localized PKA signaling in LTP, we developed a stochastic reaction-diffusion model of the signaling pathways leading to PKA activation in CA1 pyramidal neurons. Simulations investigated whether the role of anchoring is to locate kinases near molecules that activate them, or near their target molecules. The results show that anchoring PKA with adenylyl cyclase (which produces cAMP that activates PKA) produces significantly greater PKA activity, and phosphorylation of both inhibitor-1 and AMPA receptor GluR1 subunit on S845, than when PKA is anchored apart from adenylyl cyclase. The spatial microdomain of cAMP was smaller than that of PKA suggesting that anchoring PKA near its source of cAMP is critical because inactivation by phosphodiesterase limits diffusion of cAMP. The prediction that the role of anchoring is to colocalize PKA near adenylyl cyclase was confirmed by experimentally rescuing the deficit in LTP produced by disruption of PKA anchoring using phosphodiesterase inhibitors. Additional experiments confirm the model prediction that disruption of anchoring impairs S845 phosphorylation produced by forskolin-induced synaptic potentiation. Collectively, these results show that locating PKA near adenylyl cyclase is a critical function of anchoring.

## Author Summary

The hippocampus is a part of the cerebral cortex involved in formation of certain types of long term memories. Activity-dependent change in the strength of neuronal connections in the hippocampus, known as synaptic plasticity, is one mechanism used to store memories. The ability to form crisp and distinguishable memories of different events implies that learning produces plasticity of specific and distinct subsets of synapses within each neuron. Synaptic activity leads to production of intracellular signaling molecules, which ultimately cause changes in the properties of the synapses. The requirement for synaptic specificity seems incompatible with the diffusibility of intracellular signaling molecules. Anchoring proteins restrict signaling molecules to particular subcellular compartments thereby combating the indiscriminate spread of intracellular signaling molecules. To investigate whether the critical function of anchoring proteins is to localize proteins near their activators or their targets, we developed a stochastic reaction-diffusion model of signaling pathways leading to synaptic plasticity in hippocampal neurons. Simulations demonstrate that colocalizing proteins with their activator molecules is more important due to inactivation mechanisms that limit the spatial extent of the activator molecules.

## Introduction

Synaptic plasticity, the activity-dependent change in the strength of neuronal connections, is a cellular mechanism proposed to underlie memory storage. One type of synaptic plasticity is long term potentiation (LTP), which displays physiological properties that are highly suggestive of information storage. Because of the role of the hippocampus in memory, LTP in the hippocampus is studied as a model of cellular properties underlying memory [1].

The induction of long-lasting forms of LTP requires interaction among calcium-activated pathways and metabotropic-receptor-activated pathways, but the interactions among these pathways depend on the extent to which signals are spatially restricted to subcellular compartments. The production of diffusible second messengers facilitates interactions, but interferes with signaling specificity [2]. Nonetheless, an increasing number of experiments have shown that the compartmentalization of critical proteins provides downstream signaling specificity [3]. For example, a PKA-dependent form of hippocampal LTP requires not only PKA activation, but also the appropriate localization of PKA [4], [5].

Two basic mechanisms have been proposed for compartmentalization of signaling molecules: diffusional barriers and organization into multi-enzyme signaling complexes. Diffusional barriers in neurons are best exemplified by dendritic spines [6], which compartmentalize calcium due to the small size of the spine neck [7], [8]. Other synaptically activated, yet diffusible signaling molecules involved in synaptic plasticity, such as cAMP [9] and Ras [2], can spread to multiple synapses that are in close proximity on a dendrite. A second mechanism for compartmentalization is to colocalize enzymes that work together. This organization is mediated by anchoring proteins, which are structural proteins that contain binding sites for various enzymes. PKA is compartmentalized to different subcellular locations through interaction with A-Kinase Anchoring Proteins (AKAP) [10]. Binding between the PKA regulatory subunit and the AKAP produces signaling specificity of the diffusible catalytic subunit of PKA [11]. Different AKAPs, such as AKAP5, gravin, and MAP2, anchor PKA to different locations, such as to the spine or the dendrite. In addition to binding PKA, various AKAPs bind other enzymes such as adenylyl cyclase, calmodulin, phosphodiesterase, or calcineurin [12]–[14].

Though PKA-dependent LTP requires an anchored pool of PKA [4], [5], it is unknown whether the critical function of anchoring is to place PKA near adenylyl cyclase, the source of cAMP that activates PKA, or near target molecules, such as GluR1. To investigate this question, we perform simulation experiments using a novel, multi-compartment model of postsynaptic signaling pathways underlying PKA-dependent LTP in CA1 pyramidal neurons of the hippocampus. Furthermore, predictions from the model are confirmed experimentally using electrophysiological and biochemical approaches.

## Results

**Figure 1:**
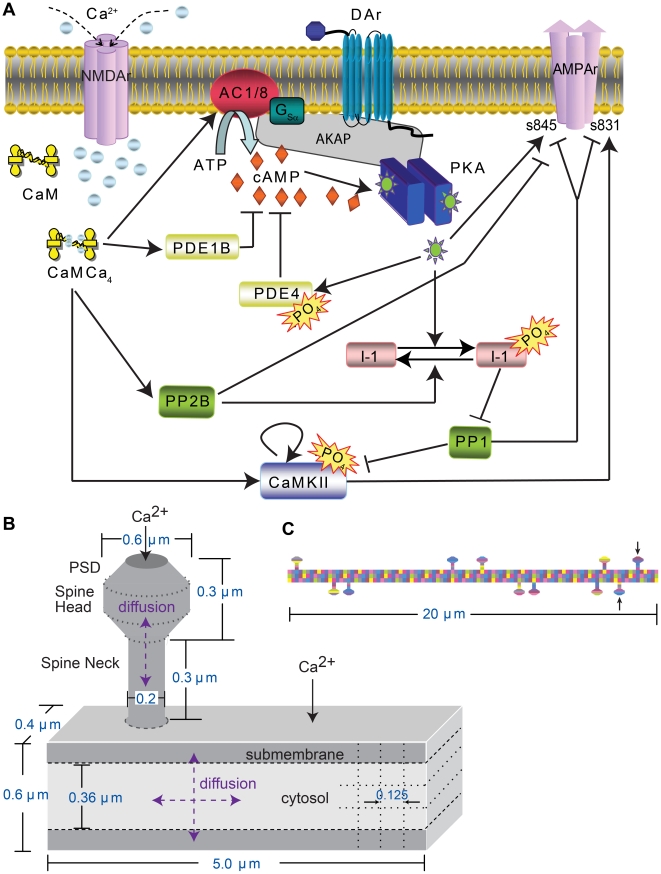
Model of CA1 pyramidal neuron dendrite plus spine. (A) Diagram of postsynaptic signaling pathways. Each arrow is modeled with one or more bimolecular or enzyme reactions. Diffusion is not illustrated in this diagram. (B) Morphology of dendrite with attached spine and location of calcium influx in the model. Dendritic subvolumes are cuboids, whereas the spine subvolumes are either cylindrical or conical, as portrayed. Dotted lines show part of the compartmentalization. Subvolumes adjacent to the top and bottom surface of the dendrite are considered submembrane subvolumes. Other dendritic subvolumes are part of the cytosol. Calcium injection in a focal dendritic region represents influx through voltage dependent calcium channels. Calcium injection in the PSD represents influx through NMDA receptors. Diffusion is two-dimensional in the dendrite and one-dimensional in the spine, with reflective boundary conditions. (C) Morphology of dendrite with multiple spines used for evaluating spatial specificity. Stimulated spines are indicated by arrows. The different colored subvolumes serve to illustrate the boundaries.

**Figure 2:**
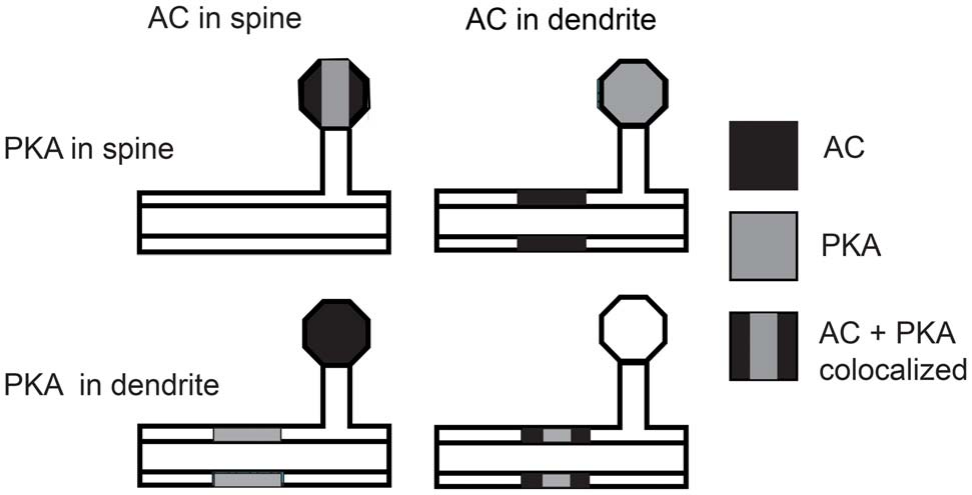
A schematic representation of the four spatial variations in location of adenylyl cyclase and PKA used for the simulations. PKA is located either in the spine head or a focal dendritic area. Similarly adenylyl cyclase (AC) is located either in the spine head or a focal dendritic area. D1R and G proteins are colocalized with AC in every case. GluR1 receptors are in the PSD compartment of the spine head for each case. Location and quantity of calcium influx (Figure 1B) is the same for all four cases.

Previous experiments showed that anchoring of PKA was necessary for LTP induced with four trains of 100 Hz stimulation applied with a 300 s interval, but they did not demonstrate whether PKA needs to be anchored near its target molecules, such as the AMPA receptor GluR1 subunit, or near a source of activator molecules, such as adenylyl cyclase that produces cAMP. Thus, to evaluate which of these two possible functions of PKA anchoring in four-train LTP is more important, the signaling pathways that underlie synaptic plasticity in hippocampal CA1 pyramidal neurons (Figure 1A) were implemented in NeuroRD [15], software for simulating stochastic reaction diffusion systems, in the morphology of a dendrite with spines (Figure 1B). Four-train LTP induction was simulated using four 1 sec trains of 100 Hz stimulation, with an 80 sec inter-train interval, which produces PKA-dependent LTP experimentally [16]. Simulations that differed only in the locations of PKA and adenylyl cyclase were compared to evaluate the two functions of molecule co-localization (Figure 2).

### Adenylyl cyclase location influences cAMP concentration and gradient

Prior to exploring PKA location, we first investigated the effect of adenylyl cyclase location on cAMP gradients. Simulations were performed with the dopamine D1 receptor and adenylyl cyclase colocalized either to the spine head or to the dendrite submembrane region, these two locations being suggested by ultrastructual analysis of dopamine receptors [17] and anchoring of adenylyl cyclase [12], [18]. G proteins were colocalized with both the receptor and the adenylyl cyclase for all simulations [19].

**Figure 3:**
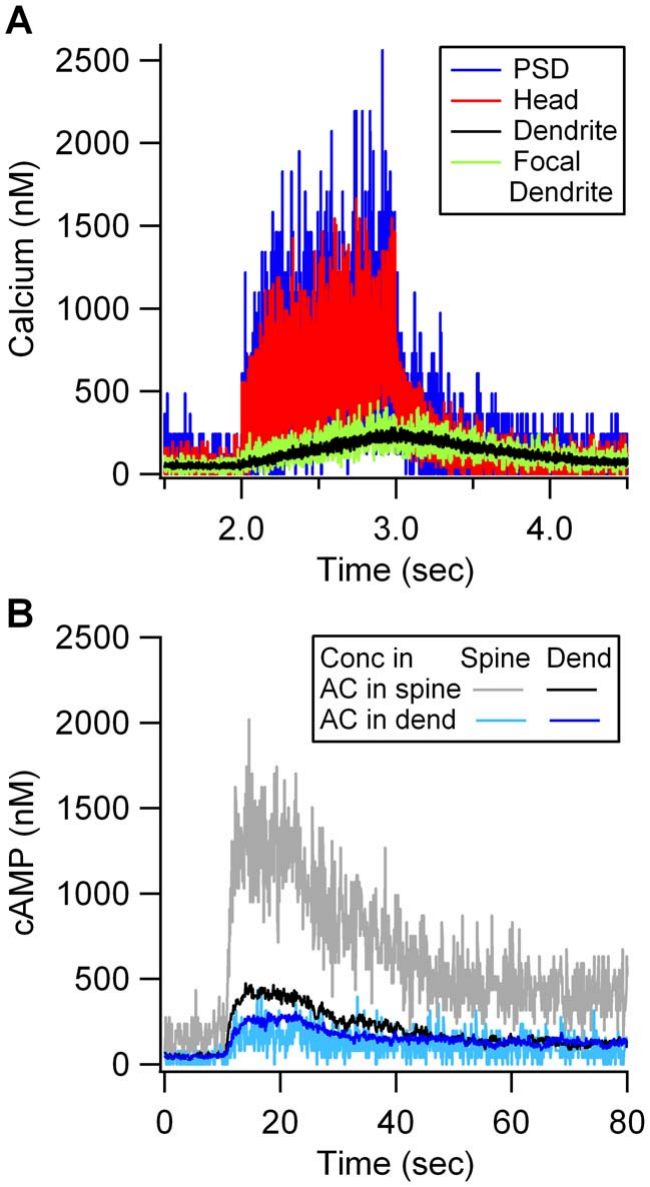
Calcium concentration leading to cAMP production, and cAMP concentration leading to PKA activity in the model. (A) Calcium gradient between spine and dendrite. Stimulation consists of 100 pulses of calcium influx (with 10 ms interval) both through the spine PSD and in a focal dendrite region. The gradient of calcium from the spine to the dendrite is similar to that measured experimentally [20]. The calcium concentration in the dendrite near the dendrite influx point, is only slightly higher than the rest of the dendrite. (B) Localization of adenylyl cyclase (AC) in the spine leads to higher cAMP concentration in the spine and a larger gradient between spine and dendrite than when adenylyl cyclase is in the dendrite. Black trace is the average cAMP concentration in the dendrite and gray trace is the average cAMP concentration in the spine when adenylyl cyclase is anchored in the spine. Dark blue is the average cAMP concentration in the dendrite and light blue is the average cAMP concentration in the spine when adenylyl cyclase is localized in the dendrite.

Simulations show that localization of dopamine D1 receptor and adenylyl cyclase in the spine leads to higher cAMP in response to stimulation (Figure 3B). Though calcium influx occurs in both spine and dendrite, calcium concentration is elevated in the spine head as compared to the dendrite (Figure 3A), similar to that measured experimentally [20]. This calcium gradient produces a greater calmodulin-activated adenylyl cyclase when it is located in the spine head as compared to the dendrite. Localizing adenylyl cyclase in the spine also produces a large gradient of cAMP from spine head to dendrite (Figure 3B). No gradient from the dendrite to the spine is apparent when adenylyl cyclase is in the dendrite, because cAMP diffuses easily to other parts of the dendrite; also, diffusion of a few molecules from the larger dendritic volume into the smaller spine volume is sufficient to raise the spine concentration.

### PKA activity depends on its location relative to adenylyl cyclase

PKA is compartmentalized to different subcellular locations through interaction with various A-kinase anchoring proteins [10]. A pool of PKA in dendritic spines is created by synaptically localized AKAPs such as AKAP5 and Yotiao [13], [21]. Recent evidence suggests that both Yotiao and AKAP5 also bind to adenylyl cyclase producing colocalization of PKA with its source of cAMP [12], [22]. Other experiments demonstrate that PKA is enriched in the dendrite via MAP2 anchoring [23]. The PKA anchored to these different locations may serve different functions.

To explore whether anchoring PKA near its activators or near its targets is more important in the induction of four-train LTP at Schaffer collateral CA1 synapses, PKA was either localized to the spine head, or placed in a focal region of the dendrite submembrane. We simulated these two spatial variations of PKA with the two spatial variations of adenylyl cyclase (Figure 2). The first case had PKA and adenylyl cyclase in the spine head, thus PKA was co-localized with both the source of cAMP and the AMPA receptor target. The second case placed PKA in the spine head but adenylyl cyclase in the dendrite submembrane, thus PKA was near the AMPA receptors but apart from adenylyl cyclase. The third case had PKA and adenylyl cyclase in the dendrite submembrane, thus PKA was co-localized with the source of cAMP, but separated from the AMPA receptors. The fourth case placed PKA in the dendrite submembrane and adenylyl cyclase in the spine head, thus PKA was apart from both its source molecules and its target. The four cases in this 2×2 experimental design (Figure 2) allowed assessment of the role of PKA location relative to its source, cAMP, or one of its targets, the AMPA receptor GluR1 subunit, separately and in combination.

**Figure 4:**
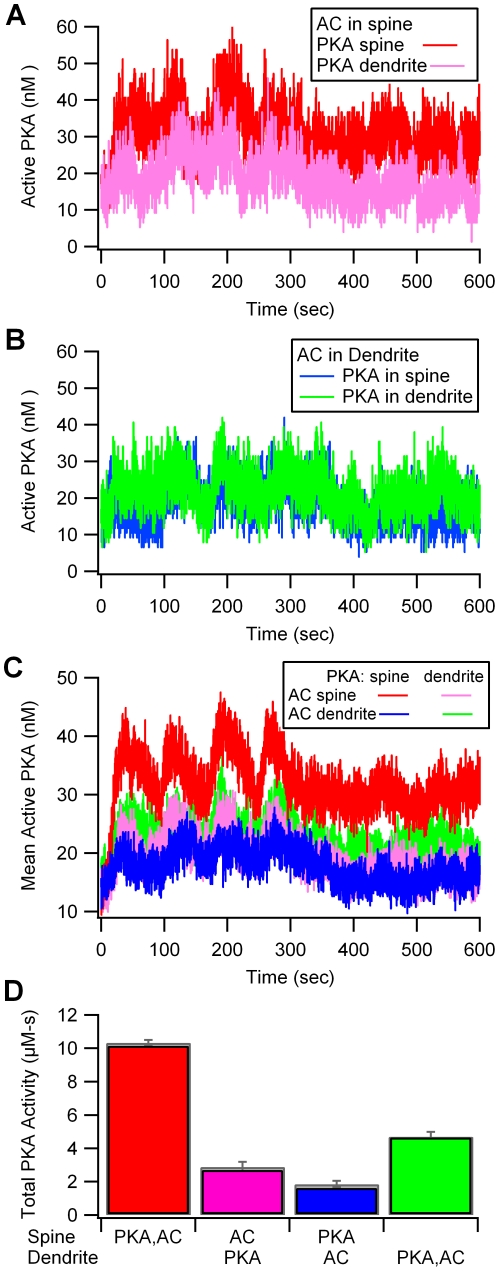
PKA activity is higher when PKA is colocalized with adenylyl cyclase (AC). (A) With AC in the spine, PKA activity is greater when PKA is anchored in the spine (red trace), than when PKA is anchored in the dendrite (pink trace). (B) With AC located in the dendrite (green and blue traces), the effect of PKA anchoring is not apparent. (C) Effect of colocalization is more apparent when averaging over five trials. The quantity of free PKA catalytic subunit is greater when PKA is colocalized with AC in the dendrite (green trace) than when PKA is separated from the AC (blue and pink traces). (D) Total PKA activity (calculated as area under the curve describing concentration of the free catalytic subunit of PKA), reveals that colocalization of AC and PKA produces significantly greater PKA activity than when AC and PKA are anchored apart.

With adenylyl cyclase located in the spine, PKA anchored in the spine produces a greater activity than PKA anchored in the dendrite (Figure 4A). The quantity of PKA catalytic subunit is small because it has high affinity for each of its many binding partners (type 4 phosphodiesterases, inhibitor-1, AMPA receptor GluR1 subunits and PKA regulatory subunits), consequently the fluctuations are large. With adenylyl cyclase located in the dendrite, the effect of PKA colocalization is not apparent (Figure 4B) because of the large fluctuations. When the results are averaged over five simulation trials (which used different random number seeds), a difference between these two cases emerges (Figure 4C). The quantity of free PKA catalytic subunit is greater when PKA is colocalized with adenylyl cylcase in the dendrite than when PKA is separated from the adenylyl cyclase. These differences due to location of PKA and adenylyl cyclase were confirmed with statistical analysis (SAS) using the procedure GLM (F = 238; P<0.0001; n = 20). Colocalizing PKA and adenylyl cyclase in the spine produces greater PKA activation than colocalizing PKA and adenylyl cyclase in the dendrite (P<0.001), which produces greater PKA activation than either of the cases with PKA and adenylyl cyclase apart from each other (P<0.001).

### The effect of PKA anchoring propagates to downstream targets

Another measure of PKA activity is phosphorylation level of downstream targets, of which four (out of many) are included in the model. Three of the PKA targets, phosphodiesterase types 4B and 4D, and inhibitor-1, are distributed throughout the neuron, whereas one of the PKA targets, the AMPA receptor GluR1 subunit, is located exclusively in the post-synaptic density of the spine. Inhibitor-1 is important because its phosphorylation level increases with LTP induction [24], and subsequent inhibition of protein phosphatase 1 enhances CaMKII phorphorylation. The AMPA receptor GluR1 subunit is phosphorylated on Ser845 by PKA, which enhances AMPA channel function and leads to increased AMPA channel expression [25], [26]. Therefore, to evaluate the role of PKA anchoring on PKA activity, we quantify the phosphorylation levels of GluR1 on Ser845, phosphodiesterase types 4B and 4D, and inhibitor-1.

**Figure 5:**
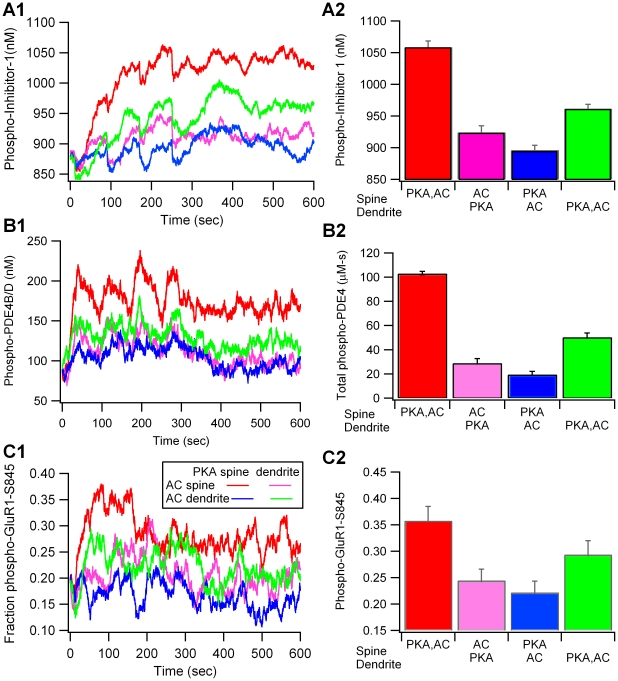
Phosphorylation of PKA targets is greatest when PKA is colocalized with cAMP production either in the spine or in the dendrite. (A1) Phospho-inhibitor-1 is greatest when PKA is colocalized with adenylyl cyclase (AC) in the spine head (red trace) and second largest when PKA is colocalized with AC in the dendrite (green trace). The early decrease in phosphorylation is caused by transient, calcium activation of calcineurin. (A2) Bar graph shows mean and s.e.m. of phospho-inhibitor-1 (n = 5 for each condition). Colocalization of AC and PKA produces significantly greater phospho-inhibitor-1 than when AC and PKA are anchored apart. (B1) Phosphorylation of PDE4s by PKA is greatest when PKA is colocalized with cAMP production either in the spine or in the dendrite. Phospho-PDE4 is the sum of phospho-PDE4B and phospho-PDE4D. (B2) The mean and s.e.m. for phospho-PDE4 represents the total activity (area under the curve) of phosphodiesterase type 4B and type 4D. (C1) Fraction of GluR1 phosphorylated on Ser845 is greatest when PKA is colocalized with both cAMP production in the spine and with the GluR1 target. (C2) The mean and s.e.m. for phospho-GluR1-S845 are calculated over 5 trials.

The location of PKA and adenylyl cyclase modulates the level of phospho-inhibitor 1 (Figure 5A; F = 68.8; P<0.0001, n = 20). The differences in phospho-inhibitor-1 levels due to anchoring are larger than the differences in free PKA catalytic subunit. When adenylyl cyclase is in the spine head, mean phospho-inhibitor-1 is greater when PKA is colocalized with adenylyl cyclase in the spine head than when PKA is in the dendrite (P<0.001). Similarly, when adenylyl cyclase is in the dendrite, mean phospho-inhibitor-1 is greater when PKA is colocalized with adenylyl cyclase in the dendrite than when PKA is in the spine (P<0.001). Thus, colocalizing PKA with its source molecules is one critical function of anchoring. Impeded diffusion of the PKA catalytic subunit from the spine to the dendrite, where most of the inhibitor-1 is located, does not prevent phosphorylation by PKA because the phosphorylation reactions are slow compared to diffusion.

Two additional PKA targets in the model are phosphodiesterase types 4B and 4D. Anchoring PKA produces a change in phosphorylation of phosphodiesterases (Figure 5B) similar to that observed with phosphorylation of inhibitor-1. Namely, colocalization of PKA with adenylyl cyclase in the spine head produces the greatest phosphorylation of phosphodiesterases. In addition, higher phosphorylation is observed when PKA is colocalized with adenylyl cyclase in the dendrite, as compared to when PKA is separated from adenylyl cyclase. Statistical analysis reveals a significant effect of PKA and adenylyl cyclase location (F = 158.6; P<0.0001; n = 20), and post-hoc tests confirm that the colocalized cases produce significantly greater phosphorylated phosphodiesterase than the non-colocalized cases (P<0.001 for each adenylyl cyclase location).

**Figure 6:**
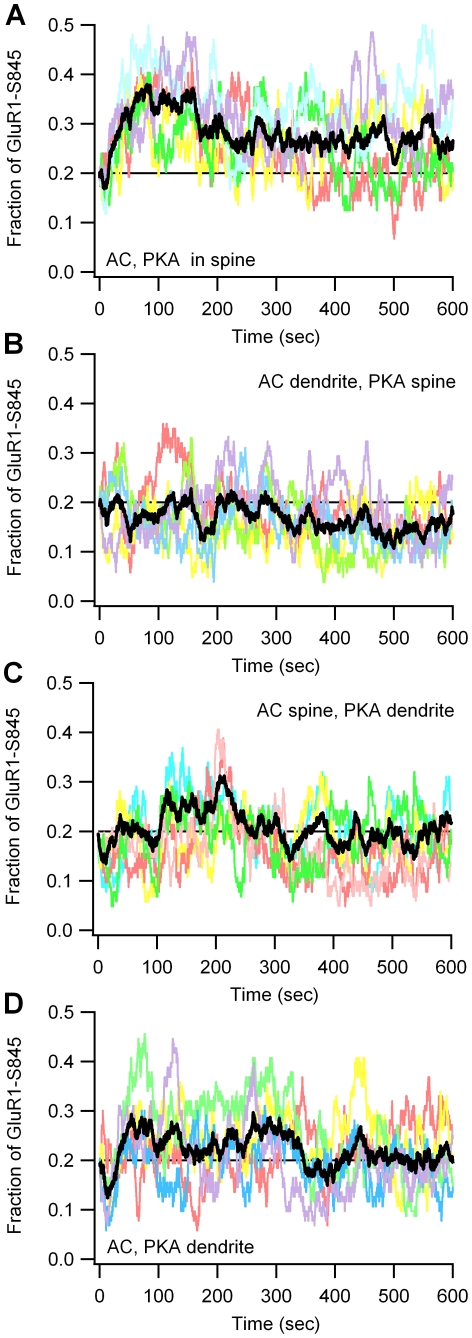
Fraction of GluR1 phosphorylated on Ser845 for 5 individual trials (shown in different colors) fluctuates due to stochastic nature of simulations. Black traces shows the average of 5 trials which differ only in the random seed used to start the simulations. (A) When PKA and adenylyl cyclase are colocalized in the spine, GluR1 phosphorylation increases for each trial. (B,C) When PKA is separated from adenylyl cyclase, most trials show a decrease in GluR1 phosphorylation; but some trials show an increase. (D) When PKA is colocalized with adenylyl cyclase in the dendrite, most trials show an increase in GluR1 phosphorylation; but some trials show a decrease.

To better assess the importance of PKA proximity to source versus target molecules, GluR1 phosphorylated on Ser845 is analyzed because it is confined to the post-synaptic density of the spine. Figure 5C illustrates that the fraction of GluR1 phosphorylated on Ser845 depends on PKA and adenylyl cyclase location (F = 9.1; P = 0.001; n = 20). Phosphorylation by PKA is greatest when PKA is colocalized both with cAMP production in the spine and with the AMPA receptor GluR1 subunit (P<0.001). More importantly, GluR1 phosphorylated on Ser845 is greater when PKA is co-localized with adenylyl cyclase in the dendrite than when PKA is apart from adenylyl cyclase but colocalized with GluR1 (P<0.05). The large fluctuations in GluR1 phosphorylation (Figure 6) suggests that not all synapses will be potentiated during experimental LTP induction. In conclusion, simulations reveal that PKA localization close to the source of cAMP is more important than localization near the target molecule.

### Robustness to parameter variations

These results are robust to variations in parameters. Diffusion constants *in vivo* are substantially slower than those measured or calculated *in vitro* [27]. To demonstrate that our results are not dependent on the estimated *in vivo* values, we repeated a subset of simulations using faster diffusion constants, closer to the *in vitro* measurements. Even with these faster diffusion constants, a greater PKA activity is observed when PKA is anchored with adenylyl cyclase in the spine, than when PKA is anchored in the dendrite away from adenylyl cyclase (Figure S1A). Because the rates for phosphorylation and dephosphorylation of GluR1 are not well constrained, we also evaluated the role of anchoring using a slower rate for dephosphorylation of phospho GluR1 by protein phosphatase 1. This change in rate constants similarly does not change the effect of colocalization on PKA activity (Figure S1B).

**Figure 7:**
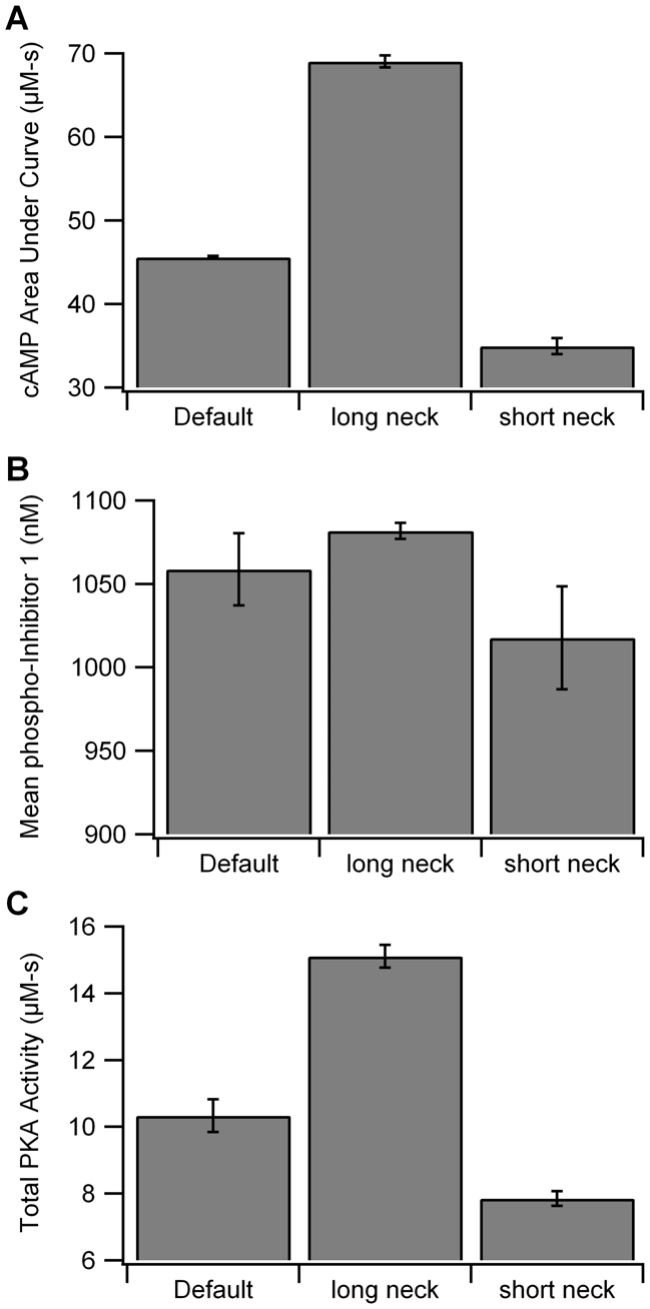
Spine neck length modulates amplitude of colocalization effect. Longer spine neck length leads to an increase in cAMP(A), which is accompanied by larger phosphorylation of inhibitor-1 (B), and greater quantity of free PKA catalytic subunit (C).

Spine morphology varies widely in hippocampal CA1 dendrites [6], and the diffusional barrier of the spine-neck geometry is an important determinant of NMDA receptor-dependent calcium signaling in the dendrite [28]. Thus, to evaluate whether spine morphology plays a role with more diffusible molecules such as cAMP, simulations were repeated using a spine with either a longer (1.0 µm) or shorter spine neck (0 µm), representing the range of experimentally measured values. The longer spine neck produces a greater cAMP concentration and larger gradient from spine to dendrite (Figure 7A). The smaller spine neck has the opposite effect, reducing the cAMP concentration. This change in cAMP due to spine neck propagates downstream to produce analogous changes in PKA activity (Figure 7B; F = 568, P<0.0001, n = 10), phosphorylation of inhibitor-1 (Figure 7C; F = 7.7; P = 0.02, n = 10) and phosphorylated PDE4s, reinforcing the role of PKA colocalization with adenylyl cyclase (as opposed to target molecules).

### Ht31 effects on PKA anchoring

Experiments show that long-lasting LTP induced with four spaced trains of synaptic stimulation is impaired in the presence of Ht31 peptide, which competes for PKA anchoring [4], [5]. To evaluate this experimental observation, we simulated PKA activity when PKA is uniformly distributed as produced by disruption of anchoring by Ht31 peptide.

**Figure 8:**
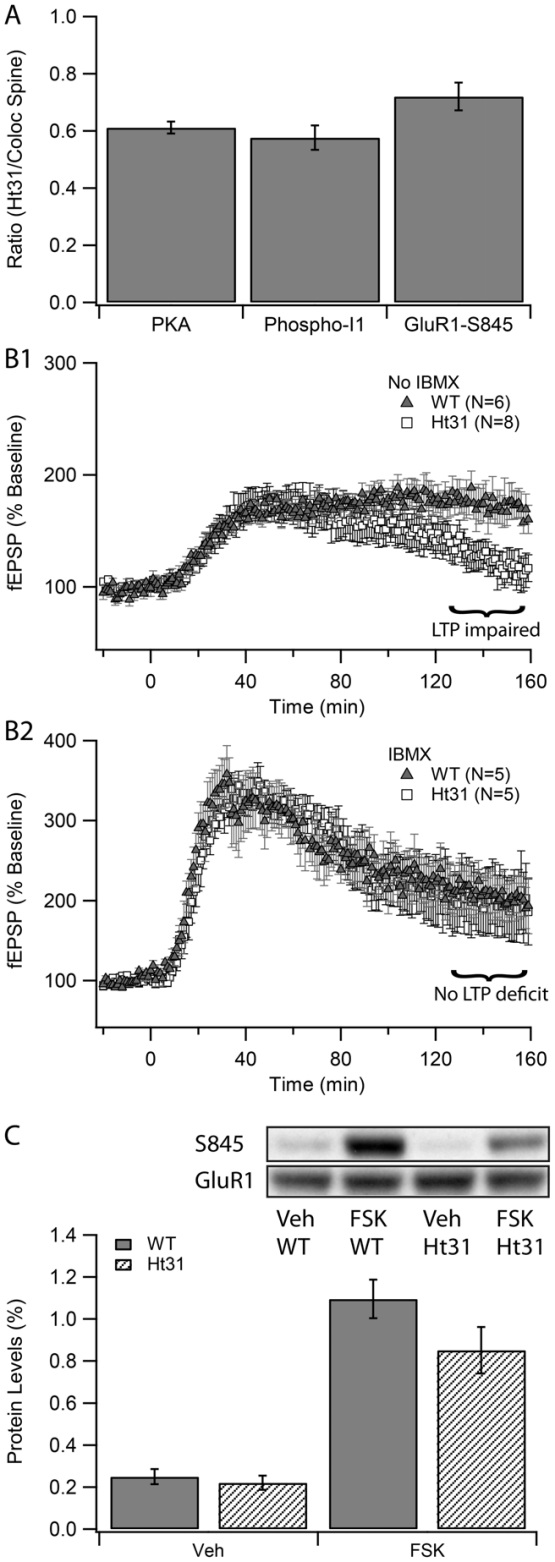
Evaluating the role of Ht31 on synaptic plasticity. (A) Ht31 disruption of PKA anchoring decreases PKA activity and phosphorylation of downstream targets in the model. The decrease in PKA activity (PKA catalytic subunit, quantity of phospho-inhibitor-1, or fraction of phospho-GluR1-S845) is quantified as ratio of those values when PKA is diffusely distributed versus colocalized with adenylyl cyclase in the spine head. (B) Experimental test of model prediction. Long-lasting synaptic potentiation is induced using forskolin (50 µM), which is delivered for 15 minutes after 20 minute baseline recording. (B1) Forskolin-induced synaptic potentiation is impaired in mice expressing Ht31. The maintenance of synaptic potentiation is impaired 2 hours after the drug treatment in Ht31 (squares) compared with wildtype (triangles) (p = 0.012). (B2) The impairment in forskolin induced potentiation is rescued in the presence of IBMX, which inhibits phosphodiesterases. There is no difference in fEPSP between Ht31 and wildtype animals 2 hours after drug treatment (p = 0.65). (C) Forskolin-induced S845 phosphorylation is impaired in mice expressing Ht31. Representative blots for S845 and GluR1 are shown at the top. The bottom graph shows the mean quantity of phosphorylated S845 on GluR1, normalized by dividing by the total GluR1 levels. Ht31 expression did not affect basal S845 phosphorylation (p = 0.79, N = 10 per genotype). In contrast, forskolin induced S845 phosphorylation was reduced in mice expressing Ht31 (p = 0.03, N = 10 per genotype).

The simulation shows that the amount of free PKA catalytic subunit, phosphorylation of inhibitor-1 and GluR1 phosphorylated on Ser845 are reduced by 30–40% (Figure 8A) when PKA is uniformly distributed. The differences between PKA colocalized with adenylyl cyclase in the spine, and PKA uniformly distributed are statistically significant (F = 256, P<0.0001 for PKA; F = 54.3, P<0.0001 for phospho-inhibitor-1; F = 11.9, P = 0.0018 for GluR1-S845; n = 14). This finding supports experimental studies showing that long-lasting LTP is blocked when anchoring is disrupted by Ht31 peptide. This finding further leads to the prediction that both phospho-inhibitor-1 and GluR1 S845 phosphorylation after experimental LTP induction will be smaller in the presence of Ht31 peptide.

Collectively, the simulation results suggest that PKA needs to be near adenylyl cyclase, to be surrounded by a high concentration of cAMP, because phosphodiesterase activity lowers the cAMP concentration as it diffuses away from the adenylyl cyclase [3]. This leads to the prediction that PDE inhibition should rescue LTP when PKA anchoring is blocked.

### Forskolin-induced synaptic potentiation and S845 phosphorylation is impaired in transgenic mice expressing Ht31

To test the computational prediction that PKA anchoring close to the source of cAMP is critical in synaptic plasticity, we use forskolin to induce synaptic plasticity in mice expressing Ht31 peptide in the hippocampus and in wildtype controls. Previous research shows that forskolin, which elevates cAMP by direct activation of adenylyl cyclase, induces PKA-dependent LTP in wildtype mice [29]. Figure 8B1 shows that forskolin-induced potentiation is impaired two hours after the drug treatment in Ht31-expressing transgenic mice (P = 0.012). The model predicts that inhibition of phosphodiesterases would rescue the LTP deficit caused by disruption of PKA anchoring. Indeed, in the presence of IBMX (3-isobutyl-1-methylxanthine), a non-specific PDE inhibitor [4], inhibition of anchoring with Ht31 peptide does not impair forskolin-induced synaptic plasticity (Figure 8B2, P = 0.65).

In a second set of slices, the quantity of phosphorylated S845 on GluR1, relative to total GluR1 levels, was measured 15 minutes after bath application with forskolin, both in wildtype and Ht31 mutant mice (Figure 8C). No difference in S845 phosphorylation was detected when slices were incubated with vehicle (P = 0.79). In contrast, the forskolin-mediated increase in GluR1 S845 phosphorylation was reduced in slices from mice expressing Ht31 (P = 0.03). Thus, as predicted by the model, disruption of PKA anchoring decreases GluR1 phosphorylation on S845 produced by activation of adenylyl cyclase.

### Spatial specificity of PKA activity

Spatial specificity of signaling and synaptic plasticity is critical for information processing, in particular for a neuron to discriminate between different patterns of input. To address whether cAMP and PKA activity exhibit spatial specificity, simulations were repeated in a 20 µm long dendrite with multiple spines (Figure 1C). Both PKA and adenylyl cyclase were colocalized in the spine head, and two of the spines, located on one end of the dendritic segment, were stimulated.

**Figure 9:**
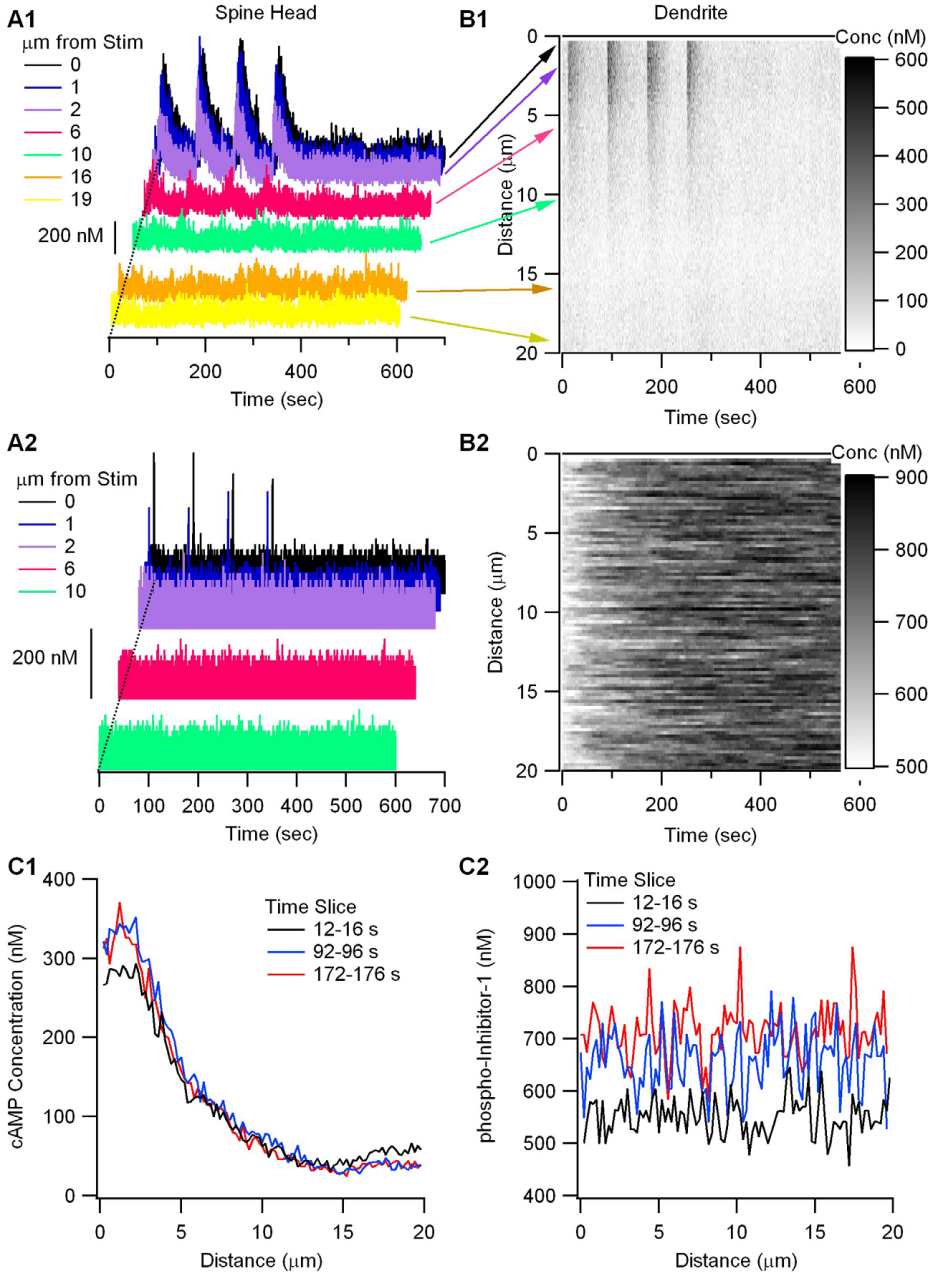
Spatial specificity of cAMP, but not PKA activity. (A) Concentration of cAMP (A1) and calcium (A2) in spines. Traces for the stimulated spines are toward the back; not all spines are illustrated. The concentration is highest in the stimulated and adjacent spines. (B) Concentration of cAMP and phospho-inhibitor versus time and space. The y-axis has been flipped, with 0 µm at the top to enhance correspondence with (A). Concentration is indicated by scale bar on the right. (B1) shows that cAMP exhibits spatial specificity. Arrows from traces in A1 to y-axis of B1 demonstrates spatial correspondence. (B2) shows that phospho-inhibitor-1 does not exhibit spatial specificity at this scale. (C) Time slices shortly after the stimulation at 10 s, 90 s and 170 s plotted against distance from the stimulated end of the dendrite. (C1) cAMP concentration exhibits spatial specificity, and does not increase with subsequent trains. (C2) Phospho-inhibitor-1 increases with subsequent trains, but does not exhibit spatial specificity.

Figure 9 shows that spatial specificity decreases as molecule activation is propagated downstream in the signaling pathways. Calcium activation in the spine head (Figure 9A2) leads to cAMP microdomains both in the spine head (Figure 9A1) and in the dendrite (Figure 9B1); however, the cAMP microdomains extend farther, e.g. 6 µm distant from the stimulated spines, than the calcium microdomains. In contrast, PKA activity does not exhibit spatial specificity in terms of phospho-inhibitor-1 (Figure 9B2) or free PKA catalytic subunit (results not shown). In addition, phospho-inhibitor-1 shows a cumulative increase with subsequent stimulus trains, though cAMP does not. This result demonstrates the importance of inactivation mechanisms for producing spatial specificity. Degradation of cAMP by phosphodiesterase prevents cAMP from diffusing throughout the dendrite, but the lack of inactivation mechanisms for PKA permits PKA to diffuse throughout the dendrite and phosphorylate inhibitor-1 far from the site of stimulation.

## Discussion

PKA is one of the key molecules in the intracellular signaling networks mediating a long lasting form of LTP in the CA1 region of the hippocampus induced by four spaced trains of high frequency stimulation. AKAPs spatially restrict PKA signaling pathways through the organization of macromolecular complexes that effectively colocalize activators and effectors of enzymes. Compartmentalization of signaling microdomains by AKAPs may be one mechanism allowing spatial specificity of LTP.

We investigated whether the critical function of AKAPs is to localize PKA near target proteins or near the source of cAMP, using a multi-compartmental stochastic reaction-diffusion model of the signaling pathways leading to PKA activation in hippocampal CA1 pyramidal neurons. Simulations show that PKA anchoring near the source of cAMP and near specific targets both enhance PKA activity; however, anchoring near the source of cAMP dominates. PKA phosphorylation of GluR1 was greater when the PKA holoenzyme was colocalized with adenylyl cylcase in the dendrite than when the PKA holoenzyme was colocalized in the spine with GluR1, but apart from the dendritic adenylyl cyclase. Experiments confirmed this model prediction by demonstrating that forskolin-induced GluR1 phosphorylation was greater in wildtype mice than in mice which express Ht31 peptide. The ideal test of the model prediction would be imaging of cAMP concentration and PKA activity simultaneously (e.g. using both an Epac FRET probe and an AKAR FRET probe [30]) during LTP induction using spatially specific synaptic stimulation. Demonstration that cAMP and PKA activity had different spatial microdomains, and that Ht31 disrupted the PKA gradient without altering the cAMP gradient awaits further development of live cell imaging techniques.

PKA anchoring near adenylyl cyclases by AKAPs is crucial for PKA signaling due to phosphodiesterases, which produce microdomains of cAMP near the adenylyl cyclase.

In other systems, phosphodiesterases produce microdomains of elevated cAMP near the adenylyl cyclase, and prevent the widespread elevation of cAMP elsewhere in the cell [15], [31], [32]. This leads to preferential activation of PKA that is colocalized with the adenylyl cyclase. The Ht31 peptide, however, disburses PKA outside the range of elevated cAMP, which results in the observed failure of long-lasting forms of LTP. Consistent with this concept, inhibition of phosphodiesterase disrupts spatial gradients of cAMP [31] and allows a cell-wide elevation of cAMP to overcome the effect of PKA mislocalization by Ht31 peptide, and rescues forskolin-induced synaptic potentiation.

The rescue of LTP with phosphodiesterase inhibitors emphasizes the importance of inactivation mechanisms as opposed to diffusional barriers for signaling specificity. The characteristic decay length of a molecule's concentration gradient is governed by the diffusion constant of the molecule as well as the inactivation rate [33], [34]; thus, gradients are stronger when the rate of inactivation is faster than the rate of diffusion. Though cAMP diffuses, increased levels of cAMP only extend to within ∼6 µm of the stimulated spines because of the strong degradation by phosphodiesterases. The PKA catalytic subunit diffuses more slowly than cAMP, yet no gradients of phospho-inhibitor-1 are observed because inactivation of PKA is rather slow, allowing it to diffuse a greater distance. Thus, this result demonstrates that also in stochastic systems the spatial gradient depends on the balance of inactivation and diffusion. Based on prior work, PKA gradients would be expected if a much longer dendrite were included in the model [35]. This suggests that spatial specificity of PKA activity on a smaller spatial scale requires additional mechanisms for PKA inactivation. The presence of a large pool of PKA anchored to MAP2 [23] available to bind to free catalytic subunit throughout the neuron may speed inactivation and provide spatial specificity of PKA activity.

Though our simulations anchored all of the PKA in a single location, these different pools of anchored PKA probably coexist in a single neuron. Diverse pools of PKA may phosphorylate different proteins in different compartments of the neuron, including the nucleus. Thus, PKA anchored in the spine may phosphorylate proteins in the spine head, of which GluR1 S845 is an example used in our simulations. Similarly, PKA anchored in the dendrite may be more important for phosphorylating molecules in the dendrite; and PKA anchored near the nucleus may be critical for control of gene transcription. The purpose of simulating the anchoring of PKA in a single location was to evaluate whether individual pools of PKA are spatially restricted. Similarly, anchoring proteins have some degree of mobility, and not all of the PKA or adenylyl cyclase is anchored to AKAPs. The mobility of the AKAP bound to both adenylyl cyclase and PKA will not alter the colocalization of PKA with adenylyl cyclase. Nonetheless, allowing for partial mobility may decrease the effect of anchoring near PKA targets in the model and may decrease the difference between colocalized and non-colocalized cases. Therefore, the rationale for completely immobilizing all of the PKA and adenylyl cyclase was to delineate which function of anchoring is most critical. One additional assumption in these simulations is that Ht31 peptide produces a uniform distribution of PKA. Imaging of PKA location in the AKAP5 knockout [36] reveals that PKA is redistributed to the soma. If this redistribution were simulated in the model by locating all the PKA at one end of the dendrite, then PKA activity in the presence of Ht31 peptide would be even lower.

The targets of PKA activity included in the model are a subset of known proteins phosphorylated by PKA. Phosphorylation of GluR1, either on S845 by PKA or on S831 by other kinases, is sufficient to support enhanced AMPA receptor conductance [26]; however, there is no evidence that GluR1 phosphorylation on Ser845 is increased after induction of LTP [37]. On the other hand, PKA phosphorylation is required for trafficking of AMPA receptors [38]. The mutation of Ser845 to Ala in GluR1 does not impair the early phase of LTP, but the effects of this mutation on long-lasting forms of LTP or forskolin-induced potentiation have not yet been examined [26]. Other PKA targets include RIM1α [39], [40], adenylyl cyclase [41], CREB [42], and constituents of the mitogen-activated-protein-kinase (MAPK) pathway. In particular, PKA phosphorylation of CREB and molecules in the MAPK pathway, either in the dendrite or in the soma and nucleus, are important for the protein synthesis and gene transcription required for long-lasting forms of LTP. Because colocalizing PKA with adenylyl cyclase enhanced the phosphorylation of *all* PKA targets in the model, it is expected that anchoring PKA with adenylyl cyclase also would enhance phosphorylation of these PKA targets needed for transcription and translation that are not included in the model.

Because the induction of LTP involves complex networks of intracellular signaling pathways, computational models have been developed to gain an understanding of events leading to LTP. Many of these models explain the temporal sensitivity of long term potentiation and depression, but very few have investigated spatial specificity or sensitivity to spatial pattern [43]. Two previous models investigated the mechanisms underlying spatial profiles of cAMP and MAPK in the dendrites. Neves et al. [35] demonstrated that both inactivation mechanisms and cell morphology contribute to the size of the cAMP and MAPK microdomains. Ajay and Bhalla [44] explained that the broad spatial profile of MAPK phosphorylation experimentally measured in the dendrite cannot be explained by diffusion, but requires distributed dendritic stimulation. Unlike the present study, neither of these models investigated the role of subcellular location of signaling proteins. Furthermore, the inclusion of spines in our model necessitates a stochastic simulation technique, because the number of molecules in the spine head is small, and inaccurately represented using concentration.

Spatial, stochastic simulations were critical to the results presented. In particular, the simulations revealed that GluR1 phosphorylation on Ser845 exhibits large fluctuations, both within trials, and between trials. In general, the stochastic fluctuations were large relative to the mean when the number of molecules was low, as compared to molecules which had a high concentration. Thus, the fluctuations in GluR1 phosphorylation on Ser845 and PKA activity were greater than the fluctuations in phospho-inhibitor 1. The large within trial variation also lead to a variation between trials: in some trials GluR1 phosphorylation on Ser845 increased, and in some trials it decreased. The average over multiple trials reduced the stochastic variation, and better represented the results that would obtain when measuring hundreds of synapses using field potentials. The variability in individual trials may correspond to the variability observed in experiments when measuring few synapses [45].

Because LTP involves spatially-restricted biochemical reactions, spatial modeling was required to investigate the effect of molecule anchoring on enzyme activation. The locally high calcium concentration in the spine was due to the diffusional barrier of the spine neck coupled with strong inactivation mechanisms. Diffusion was required for interaction between the catalytic subunit of PKA and inhibitor-1, and, in some cases, cAMP activation of PKA. Though diffusion coefficients are difficult to determine precisely (range for cAMP of 100–700 µm^2^/sec in vitro) due to the difficulty of measurements in vivo, we demonstrated that out simulation results are robust to variations of the diffusion coefficients.

Evidence suggests that anchoring of several other molecules is important for synaptic plasticity. Anchoring of calcineurin by AKAP5 plays a role in LTD [13]. Calcium-calmodulin dependent protein kinase II (CaMKII) anchors to PSD95 and either the NMDA receptor or voltage-gated calcium channels [46] depending on its state. Some experiments suggest that anchored, phosphorylated CaMKII is not accessible to dephosphorylation by protein phosphatase 1 [47]. Though this effect might play an important role in maintaining GluR1 phosphorylation on Ser 831 and controlling AMPA receptor cycling, the role of CaMKII anchoring is beyond the scope of the present research. Nonetheless, the technique and software used to investigate PKA anchoring could be applied to investigate CaMKII anchoring.

Additional evidence suggests that PKA is critical for synaptic tagging [4], [48], which provides the synaptic specificity important for information processing. The synaptic tag theory proposes that plasticity related proteins required for long-lasting forms of LTP can only be captured and utilized at synapses that have been tagged by previous activity [49]. Our results suggest that PKA anchored with adenylyl cyclase in the spine would preferentially phosphorylate tag proteins in the spine, not just GluR1. Therefore, our study of the effects of PKA spatial location on PKA activity may provide additional insights about synaptic tagging and synaptic specificity. Future simulations will explore how anchoring influences PKA phosphorylation of other substrates in other compartments, such as CREB in the nucleus, needed for production of plasticity related proteins.

## Materials and Methods

### Ethics statement

All research with animals was consistent with NIH guidelines and approved by the IACUC at the University of Pennsylvania.

### Modeling-signaling pathways

The multi-compartment, computational model (Figure 1A), consists of signaling pathways known to underlie synaptic plasticity in hippocampal CA1 pyramidal neurons. Calcium influx through the NMDA receptor leads to calcium-calmodulin activation of adenylyl cyclase types 1 and 8 [50], phosphodiesterase type 1B, protein phosphatase 2B (PP2B or calcineurin) [51] and CaMKII [52]. In addition to autophosphorylation, CaMKII can phosphorylate AMPA receptor GluR1 S831 [53], and is itself dephosphorylated by protein phosphatase 1. Dopamine binds to the G_αs_ coupled D1/D5 type receptors [54], which are expressed in CA1 [55]. G_αs_ synergistically activates adenylyl cyclase [56], which produces cAMP. After binding 4 molecules of cAMP, the two catalytic subunits (PKAc) dissociate from the regulatory subunit dimer (PKAr) and become active [57]. Targets of PKA include inhibitor-1, AMPA receptor GluR1 S845 [58], and phosphodiesterase types 4B and 4D [59]. Phosphorylated inhibitor-1 binds to and inhibits protein phosphatase 1 (PP1) [24], thereby decreasing both CaMKII and GluR1 dephosphorylation.

Because calcium is crucial for activation of adenylyl cyclase, calcium dynamics were adjusted to emulate experimental observations [7], [60], [61]. Calbindin and two submembrane calcium pumps were included to approximate calcium dynamics in the neuron [28], [62]–[65]. One pump had an affinity similar to the PMCA, the other pump had a lower affinity, similar to the NCX.

Molecules were either diffusible, non-diffusible that were evenly distributed, or non-diffusible that were anchored to specific regions. The diffusible molecules included cAMP, ATP, all forms of calmodulin, CaMKII, inhibitor-1 and the catalytic subunit of PKA. The anchored molecules included the dopamine D1 receptor, G protein, adenylyl cyclase, PKA, phosphodiesterases and AMPA receptors. Because G proteins have limited mobility in the membrane [66], they were colocalized with both the receptor and the adenylyl cyclase for all simulations, as suggested experimentally [19]. To simulated anchoring, the diffusion coefficient of anchored molecules was set to 0. The molecules were anchored in specific regions by initializing the concentration to zero in all but the anchored regions in this morphology. To specifically evaluate which function of PKA anchoring was more important (near the source of cAMP or near target proteins), D1R, G proteins, adenylyl cyclase, and PKA were anchored either in the spine head, or in a focal dendritic region which had a volume that produced a local concentration equal to that when D1R, G proteins and adenylyl cyclase were located in the spine head.

A set of chemical reactions (Tables 1–4), with concentrations of chemical species as variables, were constructed to implement these pathways in the model as illustrated in Figure 1A. Rate constants used in this model were obtained from the biochemical literature. Initial concentrations are provided in Table 5. In addition, the diffusional movements of molecules depended not only on the morphology, but also the diffusion constants, which are summarized in Table 6.

**Table 1:** Reactions and rate constants related to cAMP signaling.

Reaction Equation	k_f_ (nM^−1^ s^−1^)	k_b_ (s^−1^)	k_cat_ (s^−1^)
Da+R⇌DaR	0.0011111	10	
DaR+G_αβγ_⇌DaRG_αβγ_→DaRG_βγ_+G_α_GTP	6.0E-04	0.001	20
G_αβγ_+R⇌G_αβγ_R	6.0E-05	3.00E-04	
G_αβγ_R+Da⇌DaRG_αβγ_→DaRG_βγ_+G_α_GTP	0.0033333	10	20
DaRG_βγ_→DaR+G_βγ_	80		
G_α_GTP→G_α_GDP	10		
G_α_GDP+G_βγ_→G_αβγ_	100		
G_α_GTP+AC1⇌AC1G_α_GTP	0.0385	10	
AC1G_α_GTP+CaMCa_4_⇌AC1G_α_GTP_CaMCa_4_	0.012	0.9	
AC1G_α_GTP_CaMCa_4_+ATP⇌AC1G_α_GTP_CaMCa_4__ATP→AC1G_α_GTP_CaMCa4+cAMP	0.01	2273	28.42
AC1+CaMCa_4_⇌AC1CaMCa_4_	0.006	0.9	
AC1CaMCa_4_+ATP⇌AC1CaMCa_4__ATP→AC1CaMCa_4_+cAMP	0.01	2273	2.843
AC8+CaMCa_4_⇌AC8CaMCa_4_	0.00125	1	
AC8CaMCa_4_+ATP⇌AC8CaMCa_4__ATP→AC8CaMCa_4_+cAMP	0.01	2273	2.843
PKA+2cAMP⇌PKAcAMP_2_	8.70E-05	0.02	
PKAcAMP_2_+2cAMP⇌PKAcAMP_4_	1.15E-04	0.2	
PKAcAMP_4_⇌R2C_cAMP_4_+PKAc	0.038	0.016	
R2C_cAMP_4_⇌PKAr+PKAc	0.152	0.004	
PDE1+CaMCa_4_⇌PDE1CaMCa_4_	0.1	1	
PDE1CaMCa_4_+cAMP⇌PDE1CaMCa_4_cAMP→PDE1CaMCa_4_+AMP	0.0046	44	11
AMP→ATP	1	0	
PDE4B+cAMP⇌PDE4BcAMP→PDE4B+AMP	0.03038	77.78	19.44
PKAc+PDE4B⇌PKAcPDE4B→PKAc+*p*PDE4B	0.00428	5.6	1.25
*p*PDE4B→PDE4B	0.25		
*p*PDE4B+cAMP⇌*p*PDE4BcAMP→*p*PDE4B+AMP	0.03038	77.78	38.89
PDE4BcAMP+PKAc⇌PKAcPDE4BcAMP→*p*PDE4BcAMP+PKAc	0.00428	5.6	1.25
PDE4D+cAMP⇌PDE4DcAMP→PDE4D+AMP	0.01296	60.14	15.03
PKAc+PDE4D⇌PKAcPDE4D→PKAc+*p*PDE4D	0.00428	5.6	1.25
*p*PDE4BD→PDE4D	0.25		
*p*PDE4D+cAMP⇌*p*PDE4DcAMP→*p*PDE4D+AMP	0.01296	60.14	30.06
PDE4DcAMP+PKAc⇌PKAcPDE4DcAMP→*p*PDE4DcAMP+PKAc	0.00428	5.6	1.25
PKAcAMP_4_+PDE4B⇌PKAcAMP_4_PDE4B	6.25E-05	5.44E-03	
PKAcAMP_4_+PDE4D⇌PKAcAMP_4_PDE4D	6.25E-05	5.44E-03	
PKAcAMP_4_PDE4B⇌R2C_cAMP_4_+PKAcPDE4B	0.38	0.016	
PKAcAMP_4_PDE4D⇌R2C_cAMP_4_+PKAcPDE4D	0.38	0.016	

**Table 2 pcbi-1002084-t002:** Reactions and rate constants of calcium activated signaling pathways.

Reaction Equation	k_f_ (nM^−1^ s^−1^)	k_b_ (s^−1^)	k_cat_ (s^−1^)
CaM+2Ca⇌CaMCa_2_	0.006	9.1	
CaMCa_2_+2Ca⇌CaMCa_4_	0.1	1000	
CaM+PP2B⇌PP2BCaM	0.0046	0.0012	
PP2BCaM+2Ca⇌PP2BCaMCa_2_	0.006	0.91	
PP2BCaMCa_2_+2Ca⇌PP2BCaMCa_4_	0.1	1000	
CaMCa_2_+PP2B⇌PP2BCaMCa_2_	0.046	0.0012	
CaMCa_4_+PP2B⇌PP2BCaMCa_4_	0.046	0.0012	
CaMCa_4_+CaMKII⇌CaMKIICaMCa_4_	0.01	3	
CaMKIICaMCa_4_+CaMKIICaMCa_4_⇌Complex	1.00E-04	10	
pCaMKIICaMCa_4_+CaMKIICaMCa_4_⇌pComplex	1.00E-04	10	
pCaMKIICaMCa_4_+Complex⇌pCaMKIICaMCa_4_+pComplex	1.00E-04		
CaMKIICaMCa_4_+Complex⇌CaMKIICaMCa_4_+pComplex	1.00E-04		
Complex+Complex⇌Complex+pComplex	0.01		
Complex+pComplex⇌pComplex+pComplex	0.03		
pCaMKIICaMCa_4_⇌CaMCa_4_+pCaMKII	8.00E-04	0.01	
pCaMKII+PP1⇌pCaMKIIPP1→PP1+CaMKII	6.00E-07	0.34	0.086
pCaMKIICaMCa_4_+PP1⇌pCaMKIICaMCa_4_PP1→PP1+CaMKIICaMCa_4_	6.00E-07	0.34	0.086
I1+PKAc⇌I1PKAc→Ip35+PKAc	0.0014	5.6	1.4
I1+PKAcAMP_4_⇌I1PKA cAMP_4_	1.40E-04	5.6	
I1PKA cAMP_4_⇌R2C_ cAMP_4_+PKAcI1	0.38	0.016	
Ip35+PP1⇌Ip35PP1	0.001	0.0011	
Ip35+PP2B⇌Ip35PP2B→I1+PP2B	0.00233	11.2	2.8
Ip35PP1+PP2B⇌Ip35PP1PP2B→I1+PP1PP2B	0.00233	11.2	2.8
PP1PP2B→PP1+PP2B			1.5

**Table 3 pcbi-1002084-t003:** Reactions and rate constants of AMPA receptor pathway.

Reaction Equation	k_f_ (nM^−1^ s^−1^)	k_b_ (s^−1^)	k_cat_ (s^−1^)
GluR1+PKAc⇌GluR1−PKAc .→pS845GluR1+PKAc	0.00402	24	6
PKAcAMP_4_+GluR1⇌GluR1−PKAcAMP_4_	4.02E-04	24	
GluR1−PKAcAMP_4_⇌R2C_cAMP_4_+GluR1−PKAc	0.38	0.016	
GluR1+CaMKIICaMCa_4_⇌GluR1−CaMKIICaMCa_4_→pS831GluR1+CaMKIICaMCa_4_	2.224E-05	1.6	0.4
GluR1+pCaMKIICaMCa_4_⇌GluR1−pCaMKIICaMCa_4_→pS831GluR1+pCaMKIICaMCa_4_	2.780E-05	2	0.5
GluR1+pCaMKII⇌GluR1−pCaMKII→pS831GluR1+pCaMKII	2.224E-05	1.6	0.4
pS845GluR1+CaMKIICaMCa_4_⇌pS845GluR1−CaMKIICaMCa_4_→pS845pS831GluR1+CaMKIICaMCa_4_	2.224E-05	1.6	0.4
pS845GluR1+pCaMKIICaMCa_4_⇌pS845GluR1−pCaMKIICaMCa_4_→pS845pS831GluR1+pCaMKIICaMCa_4_	2.780E-05	2	0.5
pS845GluR1+pCaMKII⇌pS845GluR1−pCaMKII→pS845pS831GluR1+pCaMKII	2.224E-05	1.6	0.4
pS831GluR1+PKAc⇌pS831GluR1−PKAc→pS845pS831GluR1+PKAc	0.004	24	6
PKAcAMP_4_+pS831GluR1⇌pS831GluR1−PKAcAMP_4_	4.02E-04	24	
pS831GluR1−PKAcAMP_4_⇌R2C_cAMP_4_+pS831GluR1-PKAc	0.38	0.016	
pS845GluR1+PP1⇌pS845GluR1−PP1→GluR1+PP1	8.700E-04	0.68	0.17
pS845pS831GluR1+PP1⇌pS845pS831GluR1−PP1→pS831GluR1+PP1	8.750E-04	1.4	0.35
pS831GluR1+PP1⇌pS831GluR1−PP1→GluR1+PP1	8.750E-04	1.4	0.35
pS845GluR1+PP2BCaMCa_4_⇌pS845GluR1−PP2B→GluR1+PP2BCaMCa_4_	0.00201	8	2

**Table 4:** Reactions and rate constants of calcium dynamics and dopamine (L).

Reaction Equation	k_f_ (nM^−1^ s^−1^)	k_b_ (s^−1^)	k_cat_ (s^−1^)
Ca+pmca⇌pmcaCa→pmca+Ca_ext	0.05	7	3.5
Ca+ncx⇌ncxCa→ncx+Ca_ext	0.0168	11.2	5.6
Ca_ext⇌Ca	0.0017		
Ca+Calbindin⇌CalbindinCa	0.028	19.6	
Ca+CaB→CaBCa	0.028		
L⇌L_ext	2	0.000020	

To compensate for the inability to implement the known voltage dependent control of calcium dynamics that was beyond the scope of the present research, an irreversible calcium buffer (CaB) was injected after calcium influx ceased for the sole purpose of returning calcium concentration to resting level with a time course similar to experiments.

**Table 5:** Initial concentrations of molecule species in the simulation.

Molecule Name	General Cytosol (nM)
Ca	51
Ca_ext	2015100
Calbindin	149590
CalbindinCa	11348
L	10.379
L_ext	1019100
ATP	1997200
cAMP	60
PDE1	3371
PDE1CaMCa_4_	574
PDE1CaMCa_4_cAMP	3
AMP	839
CaM	9126
CaMCa_2_	315
CaMCa_4_	2
PP2BCaM	2960
PP2BCaMCa_2_	1020
PP2BCaMCa_4_	6
CaMKII	19266
CaMKII CaMCa_4_	112
pCaMKII CaMCa_4_	598
pCaMKII	26
I1	530
I1PKAc	2
Ip35	6
PP1	587
Ip35PP1	884
PDE4B	902
PDE4BcAMP	24
PKAcPDE4B	9
pPDE4B	48
PDE4D	915
PDE4DcAMP	13
PKAcPDE4D	9
pPDE4D	45

Molecule Name	PSD (nM)
pmca	65.86
pmcaCa	17.79
ncx	2996
ncxCa	157
	**Spine Cytosol** # ** (nM)**
pmca	329.28
pmcaCa	88.94
ncx	0
ncxCa	0
	**PSD (nM)**
GluR1	9756
pS845GluR	1580
pS831GluR1	279
pS845GluR -PP1	836
pS831GluR1-PP1	93

Anchored Molecules&	Spine Cytosol# (nM)	Focal Dendrite Submembrane (picoSD)*
R	1012	110
G	38427	4192
GR	6523	712
GaGTP	15	2
Gbg	483	53
AC1	34078	3717
AC1GaGTP	151	16
AC1GaGTPCaMCa_4_ATP	60	3
AC1CaMCa_4_	377	41
AC1CaMCa_4_ATP	3397	371
AC8	37098	4047
AC8CaMCa_4_	60	7
AC8CaMCa_4_ATP	649	71
PKA	18666	2698
PKAcAMP_2_	7225	1044
PKAcAMP_4_	319	46
PKAr	387	56
PKAc	365	53
R2C_cAMP_4_	205	30
PKAcAMP_4__PDE4B	160	23
PKAcAMP_4__PDE4D	205	20

Molecules not listed have initial concentrations of 0. A single molecule produces a concentration of 28 nM in the dendrite subvolumes of the single spine morphology; thus molecule concentrations less than 28 nM indicate that some subvolumes contained a single molecule and some did not, to produce the indicated concentration averaged over the entire morphology. General cytosol means that molecules populated the entire morphology.

*Molecules initialized in the dendrite submembrane are specified in picoMoles per µm^2^ (picoSD).

#Molecules initialized in the spine cytosol were excluded from the PSD, except for PKA species.

&Only one of these concentrations applied, depending on whether molecules were anchored in the spine, or in the dendrite.

**Table 6:** Diffusion constants for diffusible molecules in the model.

Molecule Name	Diffusion Constant (**µ**m^2^/sec)
Calbindin	9.3
Calbindin·Ca	9.3
CaB·Ca	10
CaB	10
Da	111.3
ATP	74.7
AMP	85.5
cAMP	86.4
CaM	11
CaMCa_2_	11
CaMCa_4_	11
CaMKII·CaMCa_4_	3.6
pCaMKII·CaMCa_4_	3.6
pCaMKII	3.6
PKA_C_	8.1
Inhibitor-1	10.6
Inhibitor-1· PKA_C_	10.6
Phospho-Inhibitor-1	10.6

Note: Molecules not listed above do not diffuse; their diffusion constants are zero.

### Modeling-morphology

The multi-compartment morphology (as default) included a 5 µm long segment of dendrite (0.6 µm wide by 0.4 µm depth) with a single spine. The spine consisted of spine head (0.6 µm diameter), neck (0.2 µm diameter and 0.3 µm long) and post-synaptic density (PSD; Figure 1B) [6]. The approximation of a cylindrical dendrite as a rectangular cuboid captured the essential axial and radial diffusion of molecules, as well as the correct surface to volume ratio.

The morphology was subdivided into multiple compartments in order to simulate the reactions and diffusion mesoscopially. The dendrite was subdivided into 200 subvolumes of dimension 0.12×0.125×0.4 µm^3^, allowing 2-dimensional diffusion The spine was subdivided into 0.1 µm cylindrical or conical slices, yielding 3 spine neck subvolumes, 2 spine head subvolumes and 1 PSD subvolume, permitting 1-dimensional diffusion. One layer of dendritic subvolumes on either edge of the dendrite was considered to be the submembrane region. The 0.12 µm width submembrane region with 0.36 µm width cytosol gave the same ratio of submembrane to cytosol volume as a cylinder with 0.07 µm width submembrane region. For simulations of the 20 µm long dendrite with 11 spines, all molecules were anchored in the spine head; thus the dendrite was subdivided into 300 subvolumes of dimension 0.2×0.2×0.4 µm^3^ and two spines located at one end of the dendrite were stimulated. Empirically, these subvolume sizes were both large enough to meet the well-stirred criterion [67] and smaller than the length scale of observed concentration gradients.

### Modeling-stimulation

Stimulation for long-lasting LTP induction consisted of four 1 sec trains of 100 Hz stimulation. Each stimulation pulse consisted of a 0.7 msec influx of calcium (62.5 molecules/msec) which accumulated during the train to approach a plateau [20]. Calcium influx occurred at two places in the model (Figure 1B): one was the PSD region and the other was the dendrite. These two areas represent influx through NMDA receptor channels [8], [68] in the spine, and voltage dependent calcium channels in the spine and dendrite [69]. An 80 sec inter-train interval was used because this interval produces PKA-dependent LTP experimentally [16]. In addition to the calcium influx, each 100 Hz train is accompanied by a 1 sec, ∼1 uM increase in dopamine [70] (0.8 molecules/msec), which binds to the G_αs_ coupled dopamine receptors.

### Modeling-numerical methods

We used a computationally efficient, Monte Carlo (stochastic) reaction-diffusion algorithm, called NeuroRD [15], for modeling these signaling pathways. The stochastic algorithm was required because many of the molecular populations were small; thus, the assumption of continuous concentration of molecules was incorrect. NeuroRD was used because the large numbers of molecules in the morphology described (Figure 1B, Table 5) made tracking individual molecules in microscopic stochastic simulators computationally prohibitive. The NeuroRD algorithm integrates the tau-leap stochastic reaction algorithm of Gillespie [71] with a computationally-efficient, stochastic diffusion algorithm [72] that, similar to tau-leap, allows multiple diffusion events at each time step. The leaping approach maintains accuracy while dramatically reducing the number of time-steps required for a simulation [73] as compared to spatial extensions of the Gillespie exact stochastic simulation algorithm [74]. Even with this accelerated algorithm, the 600 sec of simulation time (using a time step of 5 µsec) required ∼4 days for the 5 µm long dendrite and ∼8 days for the 20 µm long dendrite. Both the simulation software and the files used for the model simulations are freely available from modelDB (http://senselab.med.yale.edu/ModelDB/) and the authors website (http://krasnow.gmu.edu/CENlab/).

### Experiments

We used 3–5 months old Ht31(1) mice which express Ht31 peptide in the hippocampus [5] and wildtype littermates to verify modeling predictions. All experiments were conducted according to National Institutes of Health guidelines for animal care and use and were approved by the Institutional Animal Care and Use Committee of the University of Pennsylvania. Mice were sacrificed by cervical dislocation and hippocampi were quickly collected in ice cold, oxygenated artificial cerebrospinal fluid (aCSF) containing 124 mM NaCl, 4.4 mM KCl, 1.3 mM MgSO_4_, 1 mM NaPO_4_, 26.2 mM NaHCO_3_, 2.5 mM CaCl_2_ and 10 mM D-glucose bubbled with 95% O_2_/5% CO_2_. Transverse hippocampal slices (400 µm) were made by McIIwain tissue chopper and placed in an interface recording chamber at 28°C (Fine Science Tools, Foster City, CA). Slices were equilibrated for at least 2 hours in aCSF (pH 7.4) constantly perfused over slices at 1 ml/min. A bipolar nichrome stimulating electrode (0.5 mm; AM Systems, Carlsborg, WA) was positioned in stratum radiatum of area CA1. A glass micropipette (AM Systems, Carlsborg, WA) filled with aCSF with a resistance of 2–5 MΩ was placed next to the stimulating electrode to record field EPSPs (fEPSPs). Data were acquired using Clampex 9.2 and a Digidata 1322 A/D converter (Axon Instruments, Molecular Devices, Union City, CA) at 20 KHz and low pass filtered at 2 KHz with a 4-pole Bessel filter. Maximum 30 V stimulation was given to the stimulating electrode and slices that have maximum amplitude responses of more than 5 mV were used. The stimulus strength was set to elicit 40% of the maximum fEPSP amplitude. An adenylyl cyclase activator forskolin (Molecular grade FSK, Sigma) was prepared as a 50 µM solution and applied to slices for 15 minutes to induce chemical LTP as described before [29] and LTP was recorded for 3 hours. Synaptic strength was measured by the initial slope of fEPSP. The first 20 minute baseline values were averaged and the average was used to normalize each initial fEPSP slope.

For measurements of GluR1 phosphorylation, directly after forskolin or vehicle treatment, mouse hippocampal slices were flash frozen and stored at −80°C. Slices were lysed in buffer containing 50 mM Tris, pH 9; 1% Sodium deoxycholate, 50 mM sodium fluoride, 20 mM EDTA, 40 µM β-glycerophosphate, and 1∶100 dilutions of protease and phosphatase inhibitors. After adding NuPAGE LDS Sample Buffer (Invitrogen), 20 µg of protein was resolved using NuPAGE 4–12% Bis-Tris gels and NuPAGE MOPS Running Buffer (Invitrogen) for 2 hrs at 120 V. The separated proteins were transferred to PVDF membranes (Invitrogen) at 30 mA over night at 4°C. After blocking with 5% milk in Tris-buffered saline containing 0.1% (v/v) Tween-20 (TBST) for 1 hr with gentle shaking, membranes were incubated with antibodies directed specifically against beta-tubulin (Sigma, 1∶10,000, mouse) and phospho-S845 (Millipore, 1∶1,000, rabbit) over night at 4°C. The membranes were washed 3 times for 10 minutes in TBST. Horseradish peroxidase (HRP)-conjugated anti-rabbit or anti-mouse (Santa Cruz Biotechnology) were added 1∶1,000 in 5% milk in TBST and incubated for 2 hrs at 4°C. The membranes were washed as previously described, then incubated with Amersham ECL Western Blotting Detection Regents (GE Healthcare) for one minute. Excess ECL substrate was blotted away and the signal was detected on film (Kodak BioMax) for several time points ranging from 5 seconds to 15 minutes. Afterwards, the membranes were stripped using 10 mL Restore Western Blot Stripping Buffer (Thermo Scientific) for 20 minutes at room temperature. The membranes were washed 3 times for 10 minutes in TBST and blocked with 5% milk in TBST for 1 hr. The membranes were then incubated with total GluR1 antibody (Millipore, 1∶1000, mouse) over night at 4°C. Membranes were incubated with HRP-conjugated anti-mouse, incubated with ECL and developed following the procedure as described above. Densitometry was performed using mean gray values on ImageJ software.

### Statistical analysis

The model simulations and the role of anchoring were evaluated from the total quantity (representing both amplitude and duration of the elevation) of the enzymes PKA catalytic subunit, phosphodiesterase type 4B, and phosphodiesterase type 4D, and the mean quantity of phosphorylated inhibitor-1, and GluR1 phosphorylated on Ser845. Simulations were repeated due to stochastic variability, and the procedure General Linear Models (SAS) was employed for statistical analysis of the simulation results. In order to protect against an elevated type I error due to multiple comparisons, post-hoc tests used planned comparisons only. The effect of PKA anchoring disruption by Ht31 peptide in FSK induced chemical LTP was analyzed using the last 20 minutes of the experimental recordings. The two-sided t-test procedure was used, including tests for equality of variance, separately for the IBMX condition and for the no IBMX condition. Western blots were analyzed by first calculating the quantity of GluR1 phoshorylation on S845 relative to total GluR1, and then using the procedure General Linear Models (SAS) followed by planned contrasts. For both experiments and model simulations, data were first tested for normality using the procedure univariate (SAS), and P>0.05 was considered not significant. Both the bar graphs summarizing model simulations and the fEPSP versus time traces display mean and S.E.M.

## Supporting Information

Figure S1 Robustness of results to parameters (A) Variations in diffusion constant. (A1) cAMP concentration is greater when adenylyl cyclase is anchored in the spine (red and pink traces), than when it is anchored in the dendrite (blue and green traces). D_cAMP_ = 172.8 µm^2^/sec. (A2) PKA activity is greater when adenylyl cyclase and PKA are colocalized in the spine, similar to default cases. (B) Robustness to rate for dephosphorylation of GluR1 Ser845 by PP1. Colocalization of adenylyl cyclase with PKA still produces the greatest PKA activity (B1) and GluR1 phosphorylation on Ser845 (B2).
(JPG)
