# Supplementary figures and images for: Colocalization of Protein Kinase A with Adenylyl Cyclase Enhances Protein Kinase A Activity during Induction of Long-Lasting Long-Term-Potentiation
Source: PLoS Comput Biol. 2011 Jun 30;7(6):e1002084. doi: 10.1371/journal.pcbi.1002084 (PMC3127802; doi:10.1371/journal.pcbi.1002084)

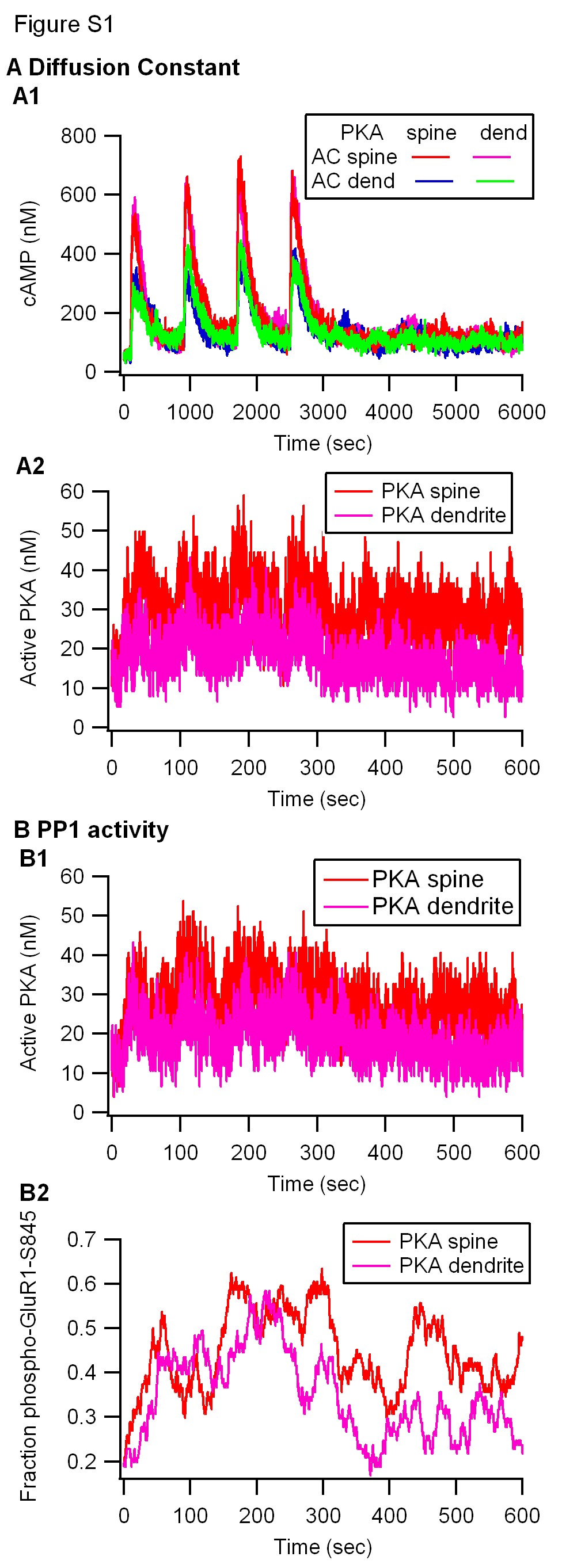

Supplement: Figure S1 — Robustness of results to parameters (A) Variations in diffusion constant. (A1) cAMP concentration is greater when adenylyl cyclase is anchored in the spine (red and pink traces), than when it is anchored in the dendrite (blue and green traces). DcAMP = 172.8 µm2/sec. (A2) PKA activity is greater when adenylyl cyclase and PKA are colocalized in the spine, similar to default cases. (B) Robustness to rate for dephosphorylation of GluR1 Ser845 by PP1. Colocalization of adenylyl cyclase with PKA still produces the greatest PKA activity (B1) and GluR1 phosphorylation on Ser845 (B2). (JPG) [file pcbi.1002084.s001.jpg]
